# The number of metabolic features as a significant prognostic factor in patients with metastatic renal cell carcinoma

**DOI:** 10.1038/s41598-020-63816-9

**Published:** 2020-04-24

**Authors:** Hyeong Dong Yuk, Eu Chang Hwang, Jae Young Park, Chang Wook Jeong, Cheryn Song, Seong Il Seo, Seok-Soo Byun, Cheol Kwak, Sung-Hoo Hong, Minyong Kang, Jinsoo Chung, Hakmin Lee

**Affiliations:** 1 Department of Urology, Seoul National University Hospital, Seoul, Korea; 2 Department of Urology, Chonnam National University Hwasun Hospital, Hwasun, Korea; 3 Department of Urology, Korea University Ansan Hospital, Korea University College of Medicine, Ansan, Korea; 4 Department of Urology, Asan Medical Center, University of Ulsan College of Medicine, Seoul, Korea; 5 Department of Urology, Samsung Medical Center, Sungkyunkwan University School of Medicine, Seoul, Korea; 6 Department of Urology, College of Medicine, The Catholic University of Korea, Seoul, Korea; 7 Department of Urology, National Cancer Center, Goyang, Korea; 8 Department of Urology, Seoul National University Bundang Hospital, Seongnam, Korea

**Keywords:** Cancer, Urology

## Abstract

The effect of metabolic characteristics on the prognosis of patients with metastatic renal cell carcinoma remains controversial. We investigated the associations between metabolic features of each individual and disease prognosis in patients with metastatic renal cell carcinoma. Data of 1,584 patients with metastatic renal cell carcinoma from a multi-institutional database were retrospectively analyzed. The entire cohort was stratified into three subgroups according to how many patients had abnormal metabolic features (hypertension, diabetes mellitus, and low body mass index). The Kaplan-Meier and Cox proportional analyses were performed to investigate the associations between abnormal metabolic features and disease prognosis. mThere were 465 subjects without any metabolic features, 995 with one or two, and 124 with three. When the survival outcomes were compared according to the number of metabolic features, patients with higher numbers of metabolic features had significantly shorter overall and cancer-specific survival than those with fewer metabolic features (all p values <0.05). The multivariate Cox analysis showed that the number of metabolic features was an independent predictor for shorter cancer-specific and overall survival (all p values < 0.05). When performing subgroup analysis according to the cellular type, significant results were only obtained among the clear cell subtype subgroup, with the association not being significant in the non-clear cell subtype cohort. Patients with more metabolic features had significantly worse survival outcomes than those with fewer metabolic features. However, the association was only statistically significant in patients with clear cell-type metastatic renal cell carcinoma.

## Introduction

Renal cell carcinoma (RCC) accounts for approximately 3–5% of the entire cancer incidence worldwide^1^. It is the sixth most frequently diagnosed malignancy in men, and the tenth commonest malignancy in women. According to the World Health Organization, >140,000 people die annually owing to RCC, which is the thirteenth commonest cause of cancer death^2^. The development of imaging technology and more frequent health check-ups have resulted in a higher incidence of RCC detection, and the early detection of renal tumors results in an overall downward stage migration^3,4^. However, a significant percentage of patients still have metastatic renal cell carcinoma (mRCC) at the time of diagnosis.3 The commonest sites of metastases are the lung, liver, bone, brain, and distant lymph nodes^5^. Approximately 40% of patients with mRCC eventually die owing to the disease, and previous studies have shown several significant associations between metabolic features and RCC^2,6–8^. Hu *et al*. demonstrated that overweight and obesity were significantly associated with higher RCC incidences in their population-based study^6^. Furthermore, Rini *et al*. found that patients with hypertension had worse prognoses during mRCC treatment^7^. From a retrospective review of a large single-center database, Psutka *et al*. showed that diabetes mellitus was independently associated with shorter cancer-specific and overall survival in patients with localized RCC after surgical treatment^8^.

Metabolic syndromes are defined as clusters of several abnormal metabolic conditions, including abdominal obesity, dyslipidemia, hyperinsulinemia, impaired fasting glucose, and high blood pressure, according to the American Heart Association^9^. A recent meta-analysis showed that metabolic syndromes were associated with an increased risk for several cancers, including liver, colorectal, and pancreatic cancers^10^. Häggström C *et al*. successfully identified a meaningful association between metabolic syndromes and RCC and found that patients with metabolic syndromes had significantly increased incidences of RCC in their large international cohort, which comprised 560,388 subjects in Europe^11^. However, the association between metabolic features and RCC prognosis has not been sufficiently assessed. Therefore, we attempted to evaluate the association between metabolic features and prognosis using a relatively large cohort of patients with mRCC.

## Methods

This retrospective study has been approved by Institutional Review Board of Seoul National University Bundang Hospital, and has been approved by all relevant institutions (B-1902-522-101), and waived the requirement to obtain informed consent from the patients. All research and related protocols used in this study complied with the principles of the Declaration of Helsinki. Data of 1,584 patients with synchronous mRCC who were diagnosed from 2003 to 2018 at nine institutions in the Republic of Korea were retrospectively analyzed. The clinical and pathologic information was acquired from our multi-institutional database, which is centrally managed. The initial staging evaluation for each patient included abdominal computed tomography (CT), chest CT, and bone scan. If needed, further work-ups, such as magnetic resonance imaging or ultrasonography, were performed. The clinical and pathologic stages were determined according to the seventh edition of the American Joint Committee on cancer TNM classification^12^. The patients’ metabolic features, including hypertension, diabetes mellitus, and low body mass index (BMI), were acquired from a review of each patient’s medical records. The cut-off for low BMI was set to 23 kg/m2 according to the Korean Society of The Study of Obesity’s definition for overweight^13^. The entire cohort was divided into three subgroups according to how many metabolic features the patients had among three metabolic features (hypertension, diabetes mellitus, low BMI) (group 1: none, group 2: one or two, group 3: three metabolic features). The patients’ mortality data were acquired from the national database of the Korean Statistical Office and our medical records. The cancer-specific and overall survivals were determined from the date of diagnosis to the date of mortality.

The independent t-test and chi-square test were performed to compare the clinical and pathologic characteristics between the groups. The Kaplan–Meier analysis with a log-rank test was utilized to evaluate the differences in survival outcomes between groups. A multivariate Cox-proportional hazard analysis was utilized to identify possible predictors of each survival outcome. SPSS software package (SPSS 20.0, Chicago, IL, USA) was utilized for all statistical analyses. All p-values were presented as two-sided values, and p < 0.05 was considered statistically significant.

## Results

**Figure 1:**
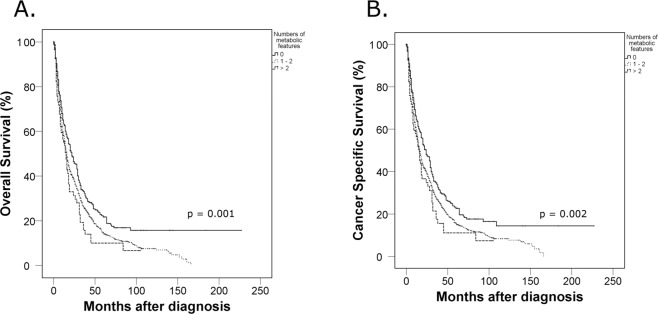
Kaplan-Meier survival curves according to the number of metabolic features on cancer-specific and overall survivals in patients with metastatic renal cell carcinoma (mRCC).

**Figure 2:**
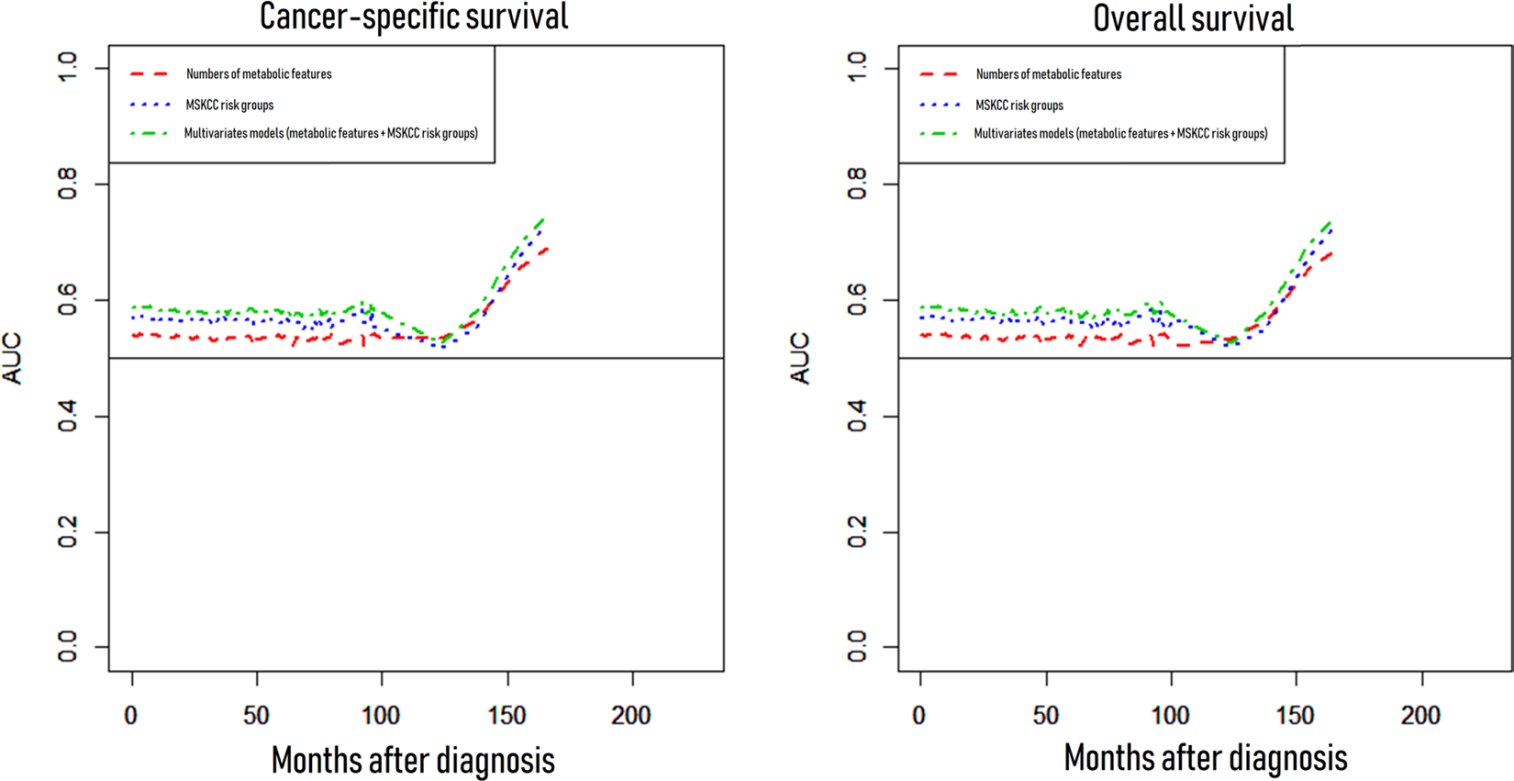
Comparison of c-indexes (numbers of metabolic features, Memorial Sloan Kettering Cancer Center risk groups, and multivariate models) on cancer-specific and overall mortality.

Clinicopathologic data of 1,584 subjects are summarized in Table 1. The median age of the patients was 60.0 years (interquartile range [IQR] 51.0–68.0), and the median survival time after diagnosis was 14.0 months (IQR 6.0–30.0). Among the patients, 964 (61.1%) underwent cytoreductive nephrectomy and 71 (4.5%) underwent metastasectomy. With respect to the location of metastases at diagnosis, 1,035 (65.3%) patients had lung metastasis, 566 (35.7%) had lymph node metastasis, 403 (25.4%) had bone metastasis, 66 (4.2%) had brain metastases, and 1,396 (88.13%) had multiple metastases at diagnosis, including metastases to the lung, liver, lymph nodes, bone, brain, soft tissue, skin, adrenal gland, gall bladder, thyroid, colon, stomach, pancreas, and parotid gland. After a median follow-up of 12.0 months (IQR 6.0–25.0), 1,164 patients died due to RCC. Overall, 1,205 subjects had all-cause mortalities after a median follow-up of 12.0 months (IQR 6.0–25.0). When we stratified patients into three subgroups according to the number of metabolic features (group 1: none, group 2: one or two, group 3: three), the Kaplan-Meier analysis revealed that there were significant differences in overall and cancer-specific survivals according to the number of metabolic features (p < 0.05) (Fig. 1) Finally, the multivariate Cox proportional analysis revealed that the numbers of metabolic features was an independent predictor for overall survival (one or two metabolic features [hazard ratio {HR}: 1.273; 95% confidence interval {CI}: 1.0652–1.534] or three metabolic features [HR: 1.423; 95% CI: 1.1172–1.811]) and cancer-specific survival (one or two metabolic features [HR: 1.434; 95% CI: 1.120–1.837] or three metabolic features [HR: 1.682; 95% CI: 1.234–2.287]) (Table 2). Moreover, the Memorial Sloan Kettering Cancer Center (MSKCC) risk factor and cytoreductive nephrectomy also showed significant associations in the multivariate analysis with cancer-specific and overall survivals. When we analyzed the c-index to evaluate the predictive ability of each variable for cancer-specific mortality and overall survival, the c-index of MSKCC criteria was 0.569 for cancer-specific mortality and 0.556 for overall mortality. In contrast, the c-indexes of the number of metabolic features were 0.538 and 0.524 for cancer-specific and overall mortality, respectively. When we combined those two variables (numbers of metabolic features, MSKCC criteria) in a multivariate model, the c-indexes of the multivariate model increased to 0.584 and 0.574 for cancer-specific and overall mortality, respectively, and these differences were statistically significant (both p value < 0.05) compared with MSKCC criteria only (Fig. 2).

**Table 1:** Patient characteristics according to the number of metabolic features.

	Entire patients (N = 1,584)	Subgroups by the number of metabolic features			p value
		0 (N = 465)	1 or 2 (N = 995)	3 (N = 124)	
Age (year)	59.1 ± 12.3	54.9 ± 13.4	59.7 ± 11.4	64.8 ± 8.9	<0.001
BMI (kg/m^2^)	23.1 ± 3.4	25.5 ± 2.3	22.6 ± 3.4	20.8 ± 1.7	<0.001
DM (yes)	326 (20.6%)	0 (0.0%)	202 (20.3%)	124 (100.0%)	<0.001
HTN (yes)	629 (39.7%)	0 (0.0%)	505 (50.8%)	124 (100.0%)	<0.001
Gender (male)	1221 (77.1%)	357 (76.8%)	775 (77.9%)	89 (71.8%)	0.528
Smoking					0.758
Non-smoker	892 (56.3%)	248 (53.3%)	563 (56.6%)	81 (65.3%)	
Ex-smoker	399 (25.1%)	125 (26.9%)	250 (25.1%)	24 (19.4%)	
Current smoker	279 (17.6%)	88 (18.9%)	173 (17.4%)	18 (14.5%)	
Unknown	14 (0.9%)	4 (0.9%)	9 (0.9%)	1 (0.8%)	
MSKCC score					0.001
Favorable	28 (1.8%)	10 (2.1%)	18 (1.8%)	0 (0.0%)	
Intermediate	931 (58.8%)	231 (49.8%)	613 (61.6%)	87 (70.2%)	
Poor	624 (39.4%)	223 (48.1%)	364 (36.6%)	37 (29.8%)	
T stage					0.140
≤T2	688 (43.4%)	201 (43.2%)	433 (43.5%)	54 (43.5%)	
≥T3	896 (56.6%)	264 (56.8%)	562 (56.5%)	70 (56.5%)	
Lymph node positive (yes)	552 (34.8%)	178 (38.3%)	331 (33.3%)	43 (34.7%)	0.081
**Site of metastasis**					
Lung	1035 (65.3%)	308 (66.4%)	640 (64.3%)	87 (70.2%)	0.356
Brain	66 (4.2%)	26 (5.6%)	36 (3.6%)	4 (3.2%)	0.226
Bone	403 (25.4%)	132 (28.4%)	247 (24.8%)	24 (19.4%)	0.706
Lymph node	566 (35.7%)	173 (37.3%)	346 (34.8%)	47 (37.9%)	0.168
Others	455 (28.7%)	147 (31.8%)	284 (28.5%)	24 (19.4%)	0.468
Cytoreductive nephrectomy (yes)	964 (60.9%)	278 (59.9%)	611 (61.4%)	75 (60.5%)	0.450

Presented by mean ± SD or numbers (percent).

**Table 2:** Multivariate Cox proportional hazards analyses of the metabolic features on overall survival and cancer specific survival.

Parameter	Overall survival		Cancer specific survival	
	HR (95% Cl)	P-value	HR (95% Cl)	P-value
Age	1.001 (0.996–1.006)	0.751	1.000 (0.995–1.005)	0.919
Smoking status		<0.001		<0.001
Non-smoker	Reference		Reference	
Ex-smoker	1.671 (0.961–2.952)	0.080	1.099 (0.957–1.263)	0.180
Current smoker	1.959 (1.092–3.494)	0.023	1.849 (1.064–3.385)	0.031
Number of metabolic features		0.010		0.016
None	Reference		Reference	
1 or 2	1.273 (1.065–1.534)	0.009	1.434 (1.120–1.837)	0.004
3	1.423 (1.117–1.811)	0.004	1.682 (1.234–2.287)	0.001
MSKCC score		<0.001		<0.001
Favorable	Reference		Reference	
Intermediate	2.846 (1.689–4.794)	<0.001	2.758 (1.636–4.648)	<0.001
Poor	4.333 (2.563–7.325)	<0.001	4.228 (2.499–7.152)	<0.001
Lymph node positive	1.103 (0.978–1.244)	0.110	1.109 (0.981–1.253)	0.098
Cytoreductive nephrectomy	0.397 (0.198–0.796)	0.009	0.364 (0.174–0.758)	0.007

**Figure 3:**
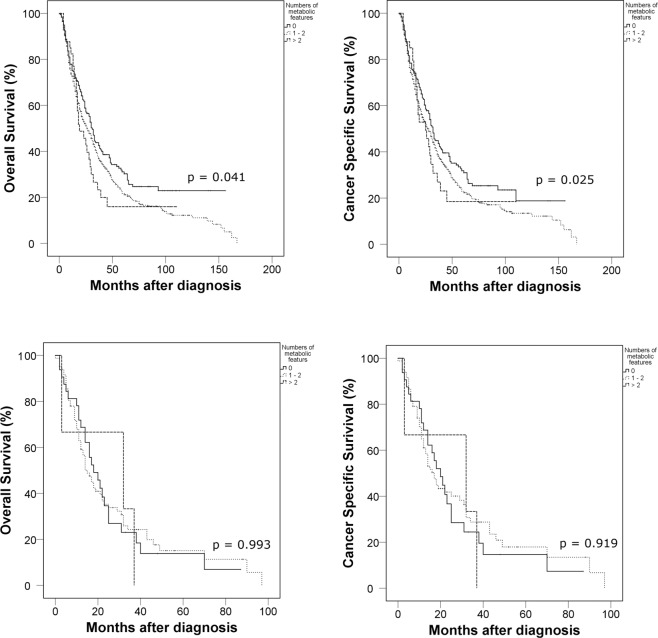
Kaplan-Meier survival curves according to the number of metabolic features on cancer-specific and overall survivals in cellular subtype subgroups (clear cell versus non-clear cell type).

Subsequently, we divided the cohort into two groups, according to cellular type (clear cell versus non-clear cell type). Patients with high numbers of metabolic features showed worse survival outcomes than patients with lower numbers of metabolic features based on the Kaplan-Meier analysis for both cancer-specific and overall survival (all p < 0.05) (Fig. 3). The high number of metabolic features was also an independent predictor for shorter overall survival (one or two metabolic features [HR: 1.322; 95% CI: 1.034–1.689] or three metabolic features [HR: 1.329; 95% CI: 1.030–1.714]) and cancer-specific survival (one or two metabolic features [HR: 1.184; 95% CI: 0.916–1.531] or three metabolic features [HR: 1.348; 95% CI: 1.049–1.733]) (Table 3). However, the patients with non-clear cell subtype did not showed any significant differences according to the number of metabolic features for both overall and cancer-specific survival (all p value> 0.05) (Fig. 3).

**Table 3:** Multivariate Cox proportional hazards analyses on overall survival and cancer specific survival in the subgroups by cellular types.

Parameter	Clear cell type				Non-clear cell type			
	Overall survival		Cancer specific survival		Overall survival		Cancer specific survival	
	HR (95% Cl)	p value	HR (95% Cl)	p value	HR (95% Cl)	p value	HR (95% Cl)	p value
Age	1.001 (0.996–1.007)	0.637	1.001 (0.995–1.006)	0.845	1.008 (0.993–1.022)	0.304	1.006 (0.991–1.022)	0.408
Smoking		0.012		0.021		0.317		0.776
Non-smoker	Reference		Reference		Reference		Reference	
Ex-smoker	1.180 (0.941–1.480)	0.152	1.183 (0.940–1.489)	0.152	0.691 (0.366–1.305)	0.254	0.801 (0.422–1.523)	0.499
Current smoker	1.707 (1.239–2.352)	0.001	1.671 (1.205–2.318)	0.002	1.359 (0.782–2.362)	0.276	1.163 (0.632–2.141)	0.627
Number of metabolic features		0.010		0.039		0.076		0.256
None	Reference		Reference		Reference		Reference	
1 or 2	1.322 (1.034–1.689)	0.026	1.184 (0.916–1.531)	0.026	3.874 (0.853–17.598)	0.079	6.164 (0.789–48.180)	0.083
3	1.329 (1.030–1.714)	0.028	1.348 (1.049–1.733)	0.020	4.928 (1.130–21.491)	0.034	7.092 (0.934–53.876)	0.058
MSKCC score		<0.001		<0.001		0.680		0.732
Favorable	Reference		Reference		Reference		Reference	
Intermediate	3.067 (1.754–5.363)	<0.001	2.987 (1.708–5.226)	<0.001	2.361 (0.313–17.832)	0.405	2.177 (0.292–16.253)	0.448
Poor	4.889 (2.788–8.574)	<0.001	4.766 (2.716–8.363)	<0.001	2.437 (0.331–17.973)	0.382	2.269 (0.297–17.355)	0.430
Lymph node positive	1.135 (1.001–1.287)	0.048	1.141 (1.004–1.296)	0.043	1.290 (0.660–2.520)	0.457	1.261 (0.712–2.233)	0.426
Cytoreductive nephrectomy	0.429 (0.378–0.486)	<0.001	0.424 (0.374–0.482)	<0.001	0.094 (0.011–0.804)	0.031	0.118 (0.014–1.029)	0.053

## Discussion

In this study, we focused on the clinical effect of metabolic characteristics in patients diagnosed with mRCC. Patients with diabetes mellitus, hypertension, or low BMI showed worse survival outcomes in terms of cancer-specific and overall survivals in our cohort (Supplementary Fig. 1). When we divided the patients according to the number of metabolic features, those with higher numbers of metabolic features showed worse oncological outcomes than those with lower numbers of metabolic features. Those associations were confined to be significant only in the subgroup with clear cell RCC subtype but not in the non-clear cell subtype cohort.

Significant associations between disease prognoses and metabolic diseases in patients with RCC have been demonstrated^8,14–16^. Psuka *et al*. investigated a large database of 1,964 subjects treated with surgery for clear cell RCC and concluded that a preoperative history of diabetes mellitus was significantly associated with postoperative oncological outcomes.8 Based on their study results, we also performed a similar study with >3,000 subjects, who were also surgical treated for localized RCC^14^. We found that preoperative diabetes mellitus was also significantly associated with shorter progression-free, cancer-specific, and overall survivals (all p < 0.05). Moreover, we found that subjects with poor glycemic control had worse oncological outcomes than those with good glycemic control, when we analyzed the subgroup with diabetes mellitus. Another study by Tsivian E *et al*. retrospectively analyzed a database of 1,748 patients with localized RCC who were treated with surgery and found a significant association between high BMI and low cellular grade^15^. They suggested that this association could be the reason why patients with RCC show an inverse association between oncological outcomes and obesity. Albiges *et al*. recently investigated a large international database of patients with mRCC and found that low BMI significantly associated with worse oncological outcomes in terms of overall survival^16^. They also analyzed the result of tissue immunohistochemistry found that the expression of fatty acid synthase was prominent in the poor and intermediate risk subgroups compared with the favorable risk group, even though the expression of fatty acid synthase was not statistically significant in their multivariate Cox-proportional analyses. Chow *et al*. analyzed the data of 389,135 subjects from the Swedish national database and found that hypertension and obesity were significantly associated with a high risk of RCC^17^.

A metabolic syndrome is defined as a combination of several metabolic conditions, such as waist circumference, high blood pressure, serum glucose, and triglycerides^18^. Approximately 24% of the population of the United States has a metabolic syndrome, which shows no discrimination among sexes^19^. Among these individuals, the Mexican- and African-American women are more likely to have metabolic syndromes than Mexican- and African-American men. Patients with metabolic syndromes are more vulnerable to cardiovascular disease than the normal population^20^. However, several epidemiologic studies have demonstrated that metabolic syndromes are also associated with a higher risk of having cancer^21–24^. Jee *et al*. analyzed a prospective database of 1,298,385 Korean subjects with 10-year follow-ups and concluded that elevated glucose levels and having diabetes mellitus were independent risk factors for having pancreatic, esophageal, colon, and liver cancers^22^. Another study from Italy also showed that diabetes mellitus and obesity were significantly associated with increased risks of certain cancers, such as liver and endometrial cancers^23^. A prospective study by Calle *et al*. examined 900,000 American adults and investigated the associations between obesity and cancer death^24^. They found that higher BMIs were associated with higher risks of mortality from every cancer, including colorectal, esophageal, liver, pancreatic, kidney, and prostate cancers. As these previous studies showed, there seems to be a significant association between metabolic condition and cancer occurrence and/or progression; however, these investigators only focused on one aspect of metabolic syndromes.

In this study, we evaluated the clinical effect of metabolic syndromes in patients with mRCC. However, our database only had information about BMI, hypertension, and diabetes mellitus, and lacked detailed data about serum lipid and glucose statuses. Therefore, we evaluated the number of metabolic features that the subjects had, rather than the exact definition of metabolic syndrome. Interestingly, we found that an increased number of metabolic features was associated with a worse prognosis. Unlike the definition of metabolic syndrome, we analyzed BMI as an inverse variable. If the patients had low BMIs and were under the cut-off for overweight, we regarded them to have metabolic features. This inverse association between obesity and prognosis of RCC patients has been previously reported and has also been referred to as the “obesity paradox”^25^. Therefore, although high BMI is the correct definition of usual metabolic syndrome, we believe that BMI has an inverse clinical effect on predicting the prognosis of patients with RCC.

Our study has some limitations. First, our results were based on the Republic of Korea’s multi-institutional data and therefore should be validated in a multi-ethnic database. Second, as a retrospective study, it had the possibility of selection or recall biases and could not determine causality. Third, data on the use of antihypertensive and diabetic drugs were not obtained. More importantly, different treatment protocols and sequencing can influence the patients’ prognosis, which was unable to be investigated in the present study due to a lack of data. However, despite these limitations, we performed a large-scale multicenter cohort analysis of mRCC and provided new clinical value on the number of metabolic features as a novel prognostic factor for predicting the oncological outcomes of patients with mRCC. When predicting the prognosis of mRCC, consideration was mainly given to performance status, hematologic status, and the time from diagnosis to treatment. However, our study found that the metabolic status-related features in patients are also worth considering in predicting the prognosis of mRCC.

## Conclusion

Patients with a higher number of metabolic features showed worse survival outcomes among patients with mRCC. These associations were only significant in the clear cell subtype and were not statistically significant in the non-clear cell subtype. These results indicate the importance of controlling related health conditions such as hypertension, diabetes, and underweight in clear cell type RCC.

## Supplementary information

Supplementary Figure 1.
