# Supplementary material for: The number of metabolic features as a significant prognostic factor in patients with metastatic renal cell carcinoma
Source: Sci Rep. 2020 Apr 24;10:6967. doi: 10.1038/s41598-020-63816-9 (PMC7181597; doi:10.1038/s41598-020-63816-9)

The number of metabolic features as a significant prognostic factor in patients with metastatic renal cell carcinoma

Authors list and Affiliations

Hyeong Dong Yuk[1], Eu Chang Hwang[2], Jae Young Park[3], Chang Wook Jeong[1], Cheryn Song[4], Seong Il Seo[5], Seok-Soo Byun[8], Cheol Kwak[1], Sung-Hoo Hong[6], Minyong Kang[5], Jinsoo Chung[7], hakmin Lee[8*]

1. Department of Urology, Seoul National University Hospital, Seoul, Korea
2. Department of Urology, Chonnam National University Hwasun Hospital, Hwasun, Korea
3. Department of Urology, Korea University Ansan Hospital, Korea University College of Medicine, Ansan , Korea
4. Department of Urology, Asan Medical Center, University of Ulsan College of Medicine, Seoul, Korea
5. Department of Urology, Samsung Medical Center, Sungkyunkwan University School of Medicine, Seoul, Korea
6. Department of Urology, College of Medicine, The Catholic University of Korea, Seoul, Korea
7. Department of Urology, National Cancer Center, Goyang, Korea
8. Department of Urology, Seoul National University Bundang Hospital, Seongnam, Korea

Sup. Figure 1. Kaplan-Meier survival curves of cancer specific and overall survivals according to (A) diabetes mellitus), (B) hypertension), and (C) body mass index >23.

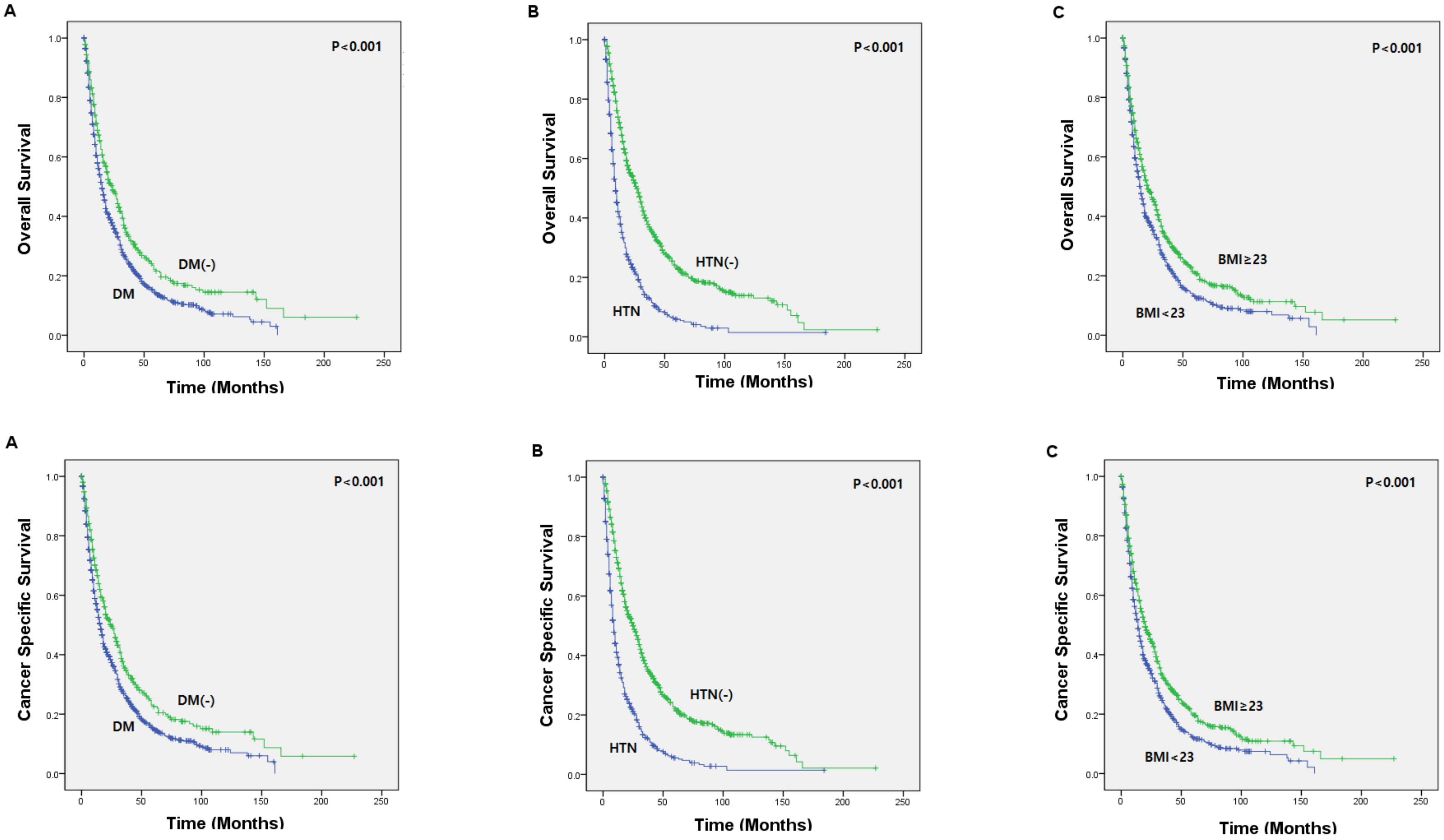

Supplement: Supplementary file 1 — Supplementary Figure 1. [file 41598_2020_63816_MOESM1_ESM.pdf]
